# Aberrant Expressions of *PSMD14* in Tumor Tissue are the Potential Prognostic Biomarkers for Hepatocellular Carcinoma after Curative Resection

**DOI:** 10.2174/0113892029277262231108105441

**Published:** 2023-12-28

**Authors:** Yi-Mei Xiong, Fang Zhou, Jia-Wen Zhou, Fei Liu, Si-Qi Zhou, Bo Li, Zhong-Jian Liu, Yang Qin

**Affiliations:** 1 Department of Biochemistry and Molecular Biology, West China School of Preclinical and Forensic Medicine, Sichuan University, Chengdu, 610 041, China;; 2 Division of Liver Transplantation, Department of Surgery, West China Hospital, Sichuan University, Chengdu, 610 041, China

**Keywords:** HCC, recurrence, prognostic biomarker, *ATP6AP1*, *PSMD14*, *HSP90AB1*

## Abstract

**Introduction:**

Hepatocellular carcinoma (HCC) has a high mortality rate, with curative resection being the primary treatment. However, HCC patients have a large possibility of recurrence within 5 years after curative resection.

**Methods:**

Thus, identifying biomarkers to predict recurrence is crucial. In our study, we analyzed data from CCLE, GEO, and TCGA, identifying eight oncogenes associated with HCC. Subsequently, the expression of 8 genes was tested in 5 cases of tumor tissues and the adjacent non-tumor tissues. Then *ATP6AP1*, *PSMD14* and *HSP90AB1* were selected to verify the expression in 63 cases of tumor tissues and the adjacent non-tumor tissues. The results showed that *ATP6AP1*, *PSMD14, HSP90AB1* were generally highly expressed in tumor tissues. A five-year follow-up of the 63 clinical cases, combined with Kaplan-Meier Plotter's relapse-free survival (RFS) analysis, found a significant correlation between *PSMD14* expression and recurrence in HCC patients. Subsequently, we analyzed the *PSMD14* mutations and found that the *PSMD14* gene mutations can lead to a shorter disease-free survival time for HCC patients.

**Results:**

The results of enrichment analysis indicated that the differentially expressed genes related to *PSMD14* are mainly enriched in the signal release pathway.

**Conclusion:**

In conclusion, our research showed that *PSMD14* might be related to recurrence in HCC patients, and the expression of *PSMD14* in tumor tissue might be a potential prognostic biomarker after tumor resection in HCC patients.

## 1 INTRODUCTION

Hepatocellular carcinoma (HCC) is the most common primary malignancy of the liver, ranking as the fifth most frequently diagnosed cancer and the third leading cause of tumor-related death worldwide [1, 2]. There are several treatment strategies available for HCC patients, including liver hepatectomy or liver transplantation, percutaneous ethanol injection (PEI), radiofrequency ablation (RFA), transarterial chemoembolization (TACE) or systemic chemotherapy. Despite liver resection being the preferred first-line therapy due to its preservation of liver function [3], the recurrence rate remains alarmingly high, with over 70% of patients experiencing a relapse within five years [4].

Vesicular or vacuolar-type adenosine triphosphatases (V-ATPases) are ATP-driven proton pumps, and their rotary motor organization closely resembles the mitochondrial ATP synthase, or FoF1 ATPase [5]. V-ATPases consist of two subcomplexes: the cytoplasmic V1 complex responsible for ATP hydrolysis and the membrane-embedded Vo complex responsible for proton transfer. *ATP6AP1* is the hub for Vo assembly [6-8]. The primary role of V-ATPases is to establish an electrochemical proton gradient across eukaryotic cell membranes, which aids in acidifying vesicles and organelles such as endosomes, lysosomes, endoplasmic reticulum, and the trans-golgi network. This process plays a role in endosome trafficking, protein degradation in lysosomes, protein glycosylation in the Golgi, neurotransmitter release, acid secretion by osteoclasts, kidney intercalated cells, and sperm maturation [5, 7-9]. Recently, the unconventional role of V-ATPases was revealed, in which *ATP6AP1* interacted with metformin-bound PEN2 to form a complex, leading to the inhibition of V-ATPase and the activation of AMPK [10-13]. V-ATPases have also been shown to be directly associated with numerous human diseases, including microbial infection, neurodegeneration, renal tubular acidosis, osteoporosis, *ATP6AP1*-CDG and cancer [5, 8-11, 13-18].

*PSMD14*, the deubiquitinase (DUB) 26S proteasome non-ATPase regulatory subunit 14, also known as RNP11 and POH1, is a subunit of the proteasome's regulatory particle. It exhibits ATP-stimulated deubiquitination directly linked to protein substrate degradation [19, 20]. *PSMD14* is highly conserved in eukaryotes from unicellular organisms to humans. It plays an important role in cellular homeostasis through regulating protein stability, aggresome clearance and disassembly, involving several biological processes, including cell cycle, senescence and stem cell renewal [21, 22]. Recently, elevated expression of *PSMD14* has been observed in various human cancers, including lung adenocarcinoma, hepatocellular carcinoma, gastric cancer, colorectal cancer, esophageal squamous cell carcinoma, breast cancer, ovarian cancer, prostate cancer, multiple myeloma, osteosarcoma, head and neck squamous cell carcinoma, and melanoma [23-34]. *PSMD14* plays an oncogenic role by stabilizing proteins, such as transcription factor AP-1, E2F1, SMAD3, EMT crucial transcription factor SNAIL,SLUG, translational regulator GRB2, BMP type 1 receptor ALK2, aerobic glycolysis enzyme PKM2, promoting cancer proliferation, metastasis and chemoresistance [24, 26, 27, 29, 33-35].

Heat shock protein 90KDA alpha, class B, member 1 (*HSP90AB1*), also known as HSP90beta, a member of the HSP family, plays a role as molecular chaperone by binding to client proteins, allowing protein folding and maintaining protein stability [36-38]. HSPs, through protein-protein interaction, bind to partners involved in various physiological processes, such as transcription, mRNA splicing, translation, cell cycle, apoptosis, intracellular transport, metabolism, autophagy, immune response, inflammation [36, 39-43]. *HSP90AB1* was reported to be associated with diseases, including Alzheimer’s disease, Parkinson’s disease, Huntington’s disease, Gaucher disease, bronchopulmonary dysplasia, glomerulosclerosis, COPD, diabetes and cancer [36, 41, 44, 45].

Cancer Cell Line Encyclopedia (CCLE, https://portals.broadinstitute.org/ccle) is a massive human cancer cell lines genome project that reflects the genetic polymorphism of human neoplasms, offering sufficient preclinical datasets for research of gene expression, mutation, and copy number variation [46]. The Gene Expression Omnibus (GEO, http://www.ncbi.nlm.nih.gov/geo/) is an international public repository where people can upload and download next-generation sequencing, microarray, and other forms of high-throughput functional genomic datasets [47, 48]. The Cancer Genome Atlas (TCGA) (http://cancergenome.nih.gov/) is an extensive dataset of cancer genome that presents diverse profiles of clinical pathological information and crucial gene transformation in 33 different kinds of cancer [49]. The datasets above broaden our horizon of human cancer and benefit the studies on neoplasms.

**Fig. (1):**
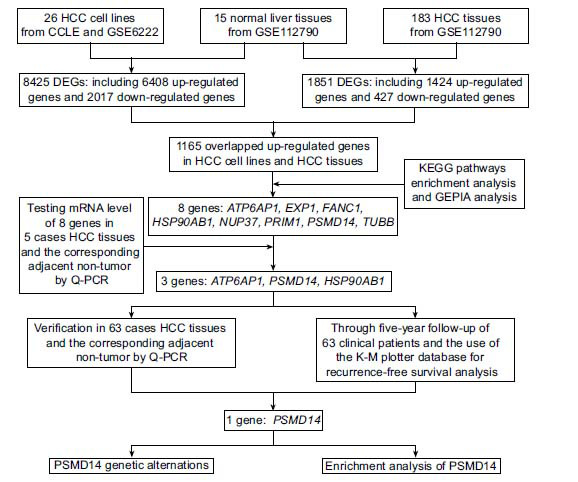
The workflow of selecting candidate genes and analyzing the relationship between the expression of candidate genes and the recurrence-free survival (RFS) for HCC patients.

For identifying prognostic biomarkers for HCC patients, we screened the oncogenes by detecting the overlapped differentiated expressed genes (DEGs) between HCC cell lines or HCC tissues and normal liver tissues by using CCLE and GEO databases. Then, KEGG pathway enrichment analysis and GEPIA platform analysis, which are based on the TCGA database, were performed to select 8 genes. In addition, the mRNA levels of 8 genes in 5 cases of HCC tissues and the adjacent non-tumor tissues were tested by quantitative polymerase chain reaction (Q-PCR). According to the results, *ATP6AP1*, *PSMD14* and *HSP90AB1,* which are generally highly expressed in HCC tissue, were chosen and verified the expressions in 63 cases of HCC tissues and the paired adjacent non-tumor tissues. Subsequently, through a five-year follow-up of 63 clinical patients and using the K-M plotter database, the relationship between relapse-free survival (RFS) analysis and the expression of *ATP6AP1*, *PSMD14* and *HSP90AB1* in HCC tissues was determined. In addition, we used the cBioPortal database to analyze the alterations of the *PSMD14* gene. Finally, we did the enrichment analysis of *PSMD14*-related differentially expressed genes (Fig. **1**).

## 2 MATERIALS AND METHODS

### 2.1 Clinical Specimens

Surgical specimens were obtained from patients with HCC who underwent liver resection at the West China Hospital in Sichuan University between 2017 and 2018. This collection included 63 tumor tissue samples and their paired adjacent non-tumor tissues, located approximately 5 cm away from the tumor. Each patient provided written informed consent for the collection of tissue specimens. This study was approved by the Institutional Review Committee of West China Hospital of Sichuan University.

### 2.2 Data Source

The original gene expression profiles of 26 HCC cell lines were extracted from the database of Cancer Cell Line Encyclopedia (CCLE) (http://www.broadinstitute.org/ccle) and GSE6222 [50] in NCBI gene expression omnibus (GEO) database (https://www.ncbi.nlm.nih.gov/geo/). GSE112790 and GSE112720 [51] original gene expression profiles comprising 15 normal liver tissue samples and 183 HCC tissue samples were downloaded from the GEO database. All microarray data of the above cell lines and tissues were based on Affymetrix Human Genome U133 Plus 2.0 Array (GPL570).

### 2.3 Data Processing

All analyses of the downloaded original gene expression profiles were performed in the R programming environment (version 3.4.3) and Bioconductor [52]. Data reading was carried out by affy R package, and then the robust multi-array average (RMA) method was used for background correction and normalization. Subsequently, the DEGs between normal liver tissues and HCC tissues or HCC cell lines, were identified through limma R package based on cut-off values: adjusted P value< 0.01 and |log fold change (FC)| > 1. The pheatmap R package was utilized to plot the heat map, and the ggplot R package was used for performing volcano plots. The Venn diagram was generated by the VennDiagram R package. Kyoto Encyclopedia of Genes and Genomes (KEGG) pathway analysis was performed to analyze the biological functions and processes of the common over-expressed DEGs in HCC cell lines and tissues, and the enrichment criterion is *p* < 0.01.

### 2.4 GEPIA (Gene Expression Profiling Interactive Analysis) Dataset

GEPIA (http://gepia.cancer-pku.cn/) is a freely available web-based tool to analyze gene expression data of 9,736 tumors and 8,587 normal samples from the Cancer Genome Atlas (TCGA) and the Genotype-Tissue Expression (GTEx) databases. The GEPIA web server features consist of seven major taps and can provide many key interactive functions that greatly facilitate data mining in cancer research, including differential expression analysis, patient survival analysis, and customizable profiling plotting [53].

### 2.5 Kaplan–Meier Plotter Database

The Kaplan-Meier plotter database sources include GEO, the European genome-phenoome Archive (EGA), and TCGA, and can assess the impact of 54,000 genes on survival in 21 cancer types (Kaplan-Meier plotter ( kmplot.com)). The Kaplan-Meier plotter was used to evaluate the prognostic value of *ATP6AP1*, *PSMD14* and *HSP90AB1* in liver cancer based on the relapse-free survival period (RFS).

### 2.6 cBioPortal Database

cBioProtal (cBioPortal for Cancer Genomics) is an open-access resource that can be used to interactively explore, analyze, and visualize multidimensional cancer genomic data [54]. We used cBioProtal analysis and evaluate the relationship between *PSMD14* gene changes and disease-free survival (DFS) of HCC patients in TCGA LIHC samples. The *p*-value of < 0.05 is considered statistically significant.

### 2.7 Quantitive PCR (Q-PCR)

Sixty-three cases of HCC tissues and the adjacent non-tumor tissues were enrolled in the Q-PCR analyses. Total RNA was extracted from tissues with a Tissue total RNA isolation kit (FOREGENE, RE-03013), and cDNA was synthesized with PrimerScript^TM^ RT reagent Kit (TAKARA,RR047A). The Q-PCR reaction conditions were listed as follows: pre-denaturation at 94°C for 3min, 30 cycles of denaturation at 94°C for 30 s, annealing at 59°C for 20 s, extension at 72°C of 30 s, and ultimate extension at 72°C of 1min. The primers for the 8 genes and GAPDH are shown in Table **1**.

### 2.8 Survival Analysis of Clinical Cases

We followed up with 63 patients who had taken specimens for a period of nearly five years (58.9 months) and obtained their relapse-free survival. According to the expression of *ATP6AP1*/*HSP90AB1*/*PSMD14* in HCC tissues, 63 patients were divided into two groups. Patients with gene expression higher than the median were in the high-expression group, and patients with gene expression lower than the median were in the low-expression group. Then we used the survival R package for survival analysis and the Kaplan-Meier survival curves were drawn by the surveminer R package.

### 2.9 Statistics Analysis

Data from the CCLE and GEO databases was processed as mentioned above. Survival analysis of RFS was conducted by using the survival R package according to the Kaplan-Meier analysis and log-rank test, and log rank *p* values ≤ 0.05 was considered statistically significant.

All these methods were performed in accordance with the relevant guidelines, regulations and the current laws for ethical conduct.

## 3 RESULTS

### 3.1 Identification of DEGs between HCC Cell Lines/HCC Tissues and Normal Liver Tissues

**Fig. (2):**
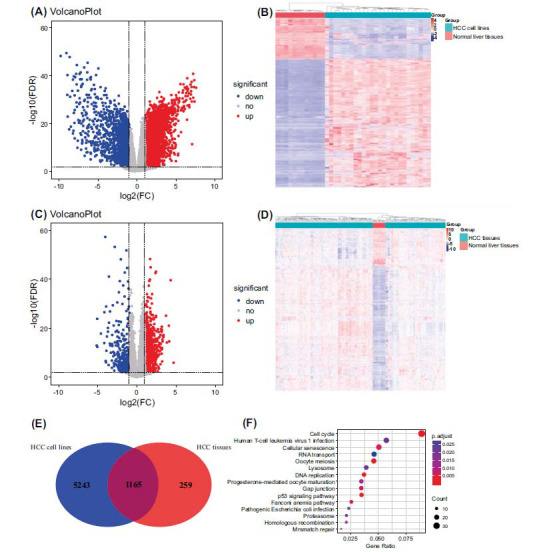
(**A**) The volcano plot of DEGs between the HCC cell lines and the normal liver tissues. The X-axis shows the fold change (log-scaled), whereas the Y-axis indicates the adjusted *p* values (log-scaled). Each symbol represents a different gene, and the red or blue colors of the symbols signify the up-regulated or down-regulated genes under threshold criteria. The fold change = 2 is set as the threshold, whereas adjust *p* value <0.01 is considered statistically significant. (**B**) The heatmap of the DEGs between HCC cell lines and normal liver tissues. Each column represented a biological sample, and each row in the heatmap represented a gene. The color indicated the expression levels of genes (6408 up-regulated and 2017 down-regulated genes). (**C**) The volcano plot of DEGs between HCC tissues and the normal liver tissues. (**D**) The heatmap of the DEGs between HCC tissues and the normal liver tissues. The color indicated the expression levels of genes (1424 up-regulated and 427 down-regulated genes). (**E**) The common up-regulated DEGs between HCC cell lines and HCC tissues. (**F**) Kyoto Encyclopedia of Genes and Genomes (KEGG) pathway enrichment analysis of the integrated DEGs.

In the present study, the gene expression profiles of 25 HCC cell lines in CCLE and one HCC cell line (Huh-7) in GSE6222 were utilized to compare gene expression with 15 normal liver tissues from GSE112790. Based on cut-off values: adjusted *P* value< 0.01 and |log fold change (FC)| > 1, the DEGs (differentiated expressed genes) were screened using the limma R package. In total, 8425 DEGs were identified, including 6408 up-regulated genes and 2017 down-regulated genes. The distribution of DEGs between HCC cell lines and normal liver tissues was visualized by a volcano plot. Red or blue dots in the plots showed significantly up-regulated or down-regulated genes respectively, and gray represented genes that express without difference (Fig. **2A**). Subsequently, a cluster heat map of DEGs was generated with R, which indicated that the gene expression patterns between HCC cell lines and normal controls were obviously distinct (Fig. **2B**). Although cancer cell lines are the workhorse of cancer research, they are neither clonal nor genetically stable and continue to evolve when cultured *in vitro* [55]. Therefore, 183 HCC tissues from GSE112790 were applied to find the common DEGs between HCC tissues and HCC cell lines. The DEGs between HCC tissues and normal liver tissues are shown in Fig. (**2C**), including 1424 up-regulated genes and 427 down-regulated genes and the cluster heatmap of DEGs is shown in Fig. (**2D**).

### 3.2 KEGG Pathway Enrichment Analysis for the Common Up-Regulated DEGs in HCC Cell Lines and HCC Tissues

As Fig. (**2E**) shows, 1165 genes were observed up-regulated and overlapped in HCC cell lines and HCC tissues. Except for seven genes that don’t have the corresponding ENTREZ ID, the other common up-regulated DEGs were enriched by KEGG pathway enrichment analysis to understand their function in HCC further. According to the enrichment criterion *p* < 0.01, sixteen KEGG pathways were selected, including Cell cycle, DNA replication, p53 signaling pathway, Oocyte meiosis, Cellular senescence, Gap junction, Fanconi anemia pathway, Mismatch repair, Homologous recombination, Progesterone-mediated oocyte maturation, Lysosome, Human T-cell leukemia virus 1 infection, Proteasome, Pathogenic *Escherichia coli* infection, and RNA transport (Table **2** and Fig. **2F**). However, a number of DEGs were not enriched for any pathways.

### 3.3 Identification of 8 Oncogenes through the Enriched KEGG Pathways and GEPIA

**Fig. (3):**
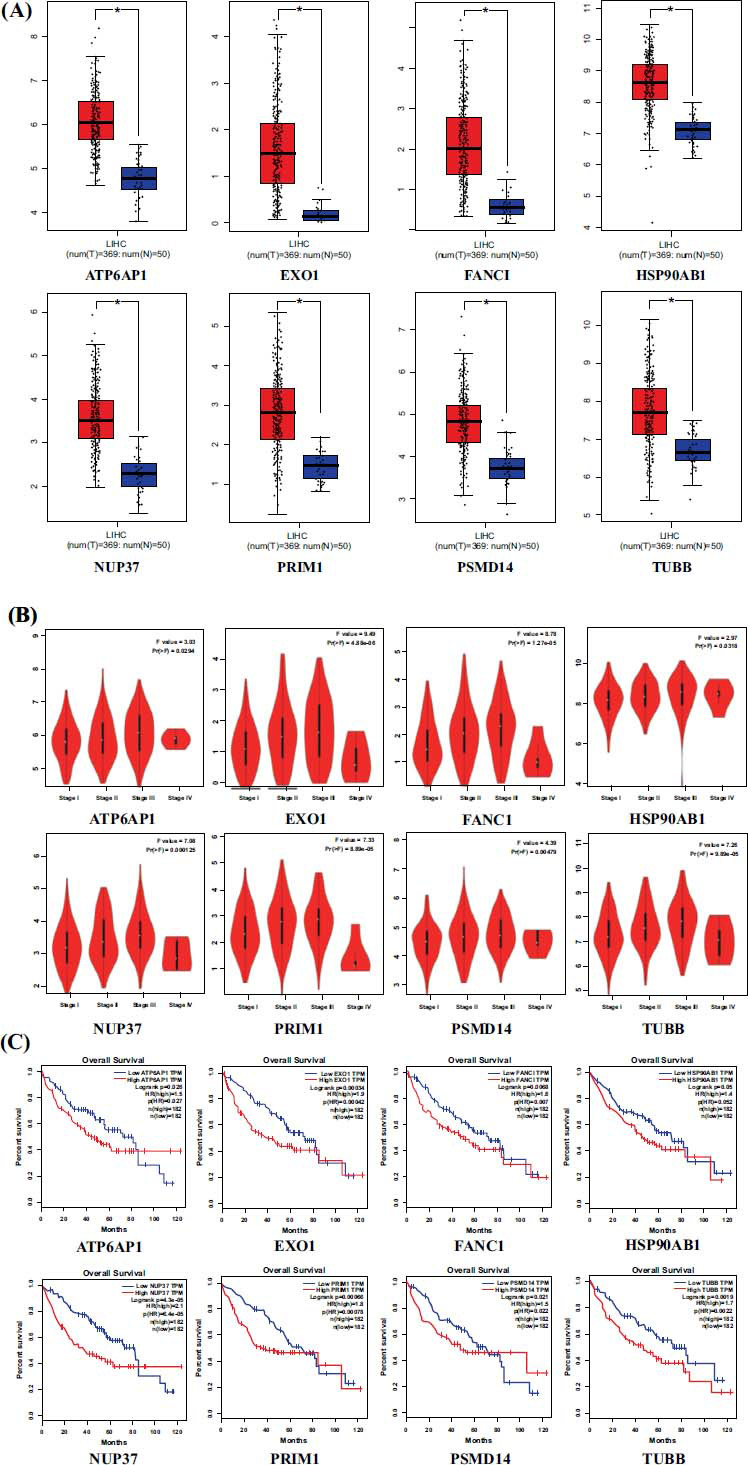
The analysis results of *AP6AP1, EXO1, FANCI, HSP90AB1, NUP37, PRIM1, PSMD14 and TUBB* in HCC through GEPIA. (**A**) The expression levels of the 8 genes in HCC tissues and normal liver tissues. (**B**) The correlation between the expression status of 8 genes and the tumor stage. (**C**) The Kaplan-Meier survival curves of OS rates for the 8 genes in HCC patients.

With the hope of finding new prognostic biomarkers for HCC, GEPIA (Gene Expression Profiling Interactive Analysis) platform based on TCGA database was performed to analyze the oncogenes in each enriched KEGG pathway. Eight genes were selected, consisting of *ATP6AP1, EXO1, FANCI, HSP90AB1, NUP37, PRIM1, PSMD14* and *TUBB* (Table **3**). The expression levels of these genes in HCC tissues and normal liver tissues are shown in Fig. (**3A**). The results revealed that all of the above genes presented a significantly higher expression in HCC tissues (all *p* < 0.05). In addition, the expression of these genes was closely associated with the tumor stage (Fig. **3B**, all *p* < 0.05). The Kaplan–Meier survival curves also demonstrated that the high expression of the 8 genes was significantly correlated with the poor overall survival (OS) of HCC patients: *ATP6AP1* (logrank *p* = 0.026, HR(high)=1.5), *EXO1* (logrank *p* = 0.00034, HR(high)=1.9), *FANCI* (logrank *p* = 0.0066, HR(high)=1.6), *HSP90AB1* (logrank *p* = 0.05, HR(high)=1.4), *NUP37* (logrank *p* = 4.31e-05, HR(high)=2.1), *PRIM1* (logrank *p* = 0.00066, HR(high)=1.8), *PSMD14* (logrank *p* = 0.021, HR(high)=1.5), *TUBB* (logrank *p* = 0.0019, HR(high)=1.7) (Fig. **3C**).

## 4 Q-PCR TESTED THE EXPRESSION OF THE 8 GENES IN HCC TISSUES AND PAIRED ADJACENT NON-TUMOR TISSUES, AND THE 3 CANDIDATE ONCOGENES WERE SELECTED

**Fig. (4):**
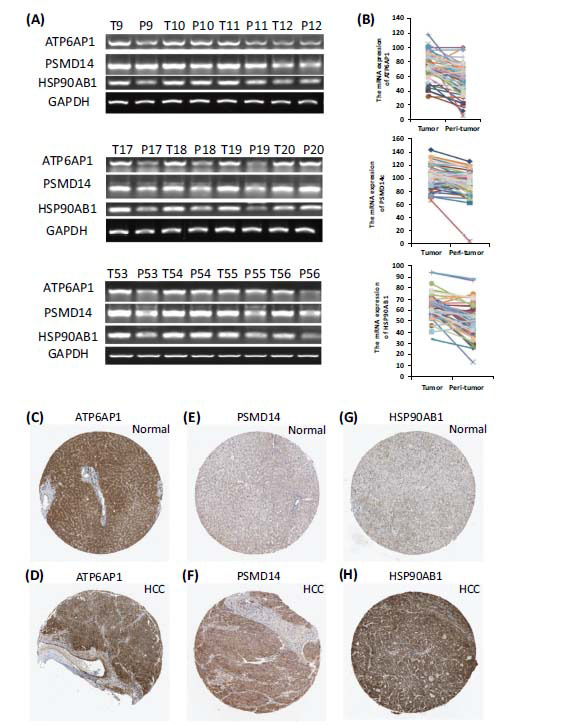
(**A, B**) The mRNA levels of *ATP6AP1*, *PSMD14* and *HSP90AB1* in 63 cases of HCC tissues and the corresponding adjacent non-tumor tissues. Immunohistochemical results of the expression of *ATP6AP1* (**C, D**), *PSMD14* (**E, F**), and *HSP90AB1* (**G, H**) proteins in normal liver and HCC tissues in the HPA database.

To further explore the role of 8 oncogenes in HCC, the mRNA expression of the 8 oncogenes was detected by Q-PCR in 5 cases of HCC tissues and corresponding adjacent non-tumor tissues. We found that only *ATP6AP1, PSMD14* and *HSP90AB1*, were generally existent and highly expressed in tumor tissue (data not shown). Then, we selected *ATP6AP1*, *PSMD14* and *HSP90AB1* as the candidate genes and verified the expressions in 63 cases of tumor tissues and the adjacent non-tumor tissues. We identified *ATP6AP1, PSMD14* and *HSP90AB1*, which were exactly highly expressed in tumor tissue (Fig. **4A-D**). In addition, the immunohistochemical analysis obtained in the Human Protein Atlas database (HPA) showed that the expression level of *PSMD14* (Fig. **4E**, **F**), *HSP90AB1* (Fig. **4G**, **H**) in HCC tissue is higher than that of normal liver tissue, but the expression difference of *ATP6AP1* is not sufficiently pronounced.

## 5 SURVIVAL ANALYSIS OF *ATP6AP1, HSP90AB1, AND PSMD14* IN THE HCC TISSUES THROUGH DATABASE AND CLINICAL CASE FOLLOW-UP

To assess the relationship between *ATP6AP1* , *PSMD14* , and *HSP90AB1* and hepatocellular carcinoma, we used the Kaplan-Meier Plotter for prognostic survival analysis. Fig. (**5A**) demonstrates that in HCC, a high expression of *ATP6AP1* (log rank *p* = 0.11) did not significantly impact patient RFS. However, as depicted in Fig. (**5B** and **C**), elevated expression levels of *PSMD14* (log rank *p* = 0.00063) and *HSP90AB1* (logrank *p* = 0.05) were significantly and negatively associated with patient RFS.

Additionally, we conducted a nearly five-year follow-up on 63 clinical patients from whom tissue samples had been previously collected; data from one patient was lost. As shown in Fig. (**5D** and **F**), there is no significant correlation between *ATP6AP1* (logrank *p* = 0.39), *HSP90AB1* (logrank *p* = 0.90) and RFS. As shown in Fig. (**5E**), the analysis results of *PSMD14* are consistent with the results of the K-M plotter that high expression is negatively correlated with RFS (logrank *p* = 0.043). While the variance in the *ATP6AP1* analysis wasn't statistically significant (*P* > 0.05), we noted a clear distinction between the curves of the high-expression and low-expression groups. Observationally, higher *ATP6AP1* expression seemed to correlate with a higher patient recurrence rate.

## 6 GENETIC ALTERATIONS OF *PSMD14* ARE ASSOCIATED WITH DISEASE-FREE SURVIVAL IN HCC PATIENTS

**Fig. (6):**
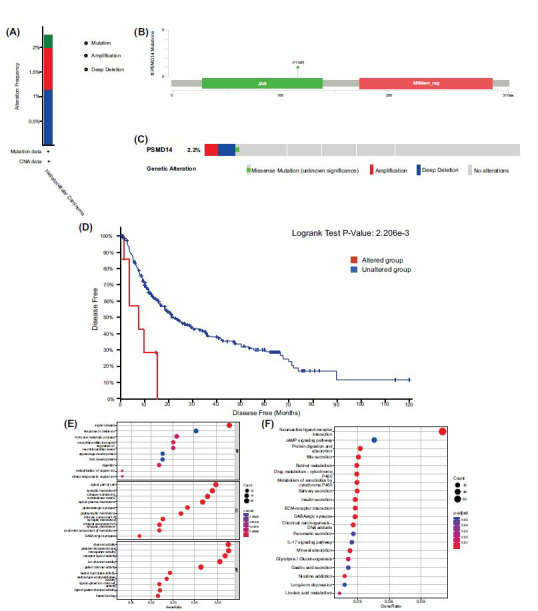
(**A**) The type and frequency of *PSMD14* mutations in HCC. (**B**) Mutation site of *PSMD14*. (**C**) Frequency of *PSMD14* mutation. (**D**) Mutations in *PSMD14* were associated with shorter DFS time (*p* <0.05) in HCC patients. (**E**) The significantly enriched GO annotations in HCC. (**F**) The KEGG pathways of *PSMD14* in HCC. BP, Biological Process; CC, Cellular component; MF, Molecular Function.

We analyzed the *PSMD14* gene mutations in HCC samples from the TCGA (Firehose Legacy, n = 379) using the cBioPortal database. The data revealed that 2.12% of HCC patients had mutations in the *PSMD14* gene (Fig. **6A** and **C**), with deep deletion being the most frequent mutation type. A specific mutation site on *PSMD14* is shown in Fig. (**6B**). Patients were grouped based on the presence or absence of *PSMD14* gene mutations. Using the Kaplan-Meier survival curve and the log-rank test, we found a significant association between disease-free survival (DFS) and *PSMD14* mutations ( *p* < 0.05). Notably, the DFS duration for the mutant group was considerably shorter than that for the non-mutant group (Fig. **6D**).

## 7 ANALYSIS OF GO AND KEGG PATHWAYS ASSOCIATED WITH *PSMD14* IN HCC

Subsequently, we annotated the major gene ontology (GO) of *PSMD14*-related differentially expressed genes through gene set enrichment analysis (GSEA) (Fig. **6E**). These differentially expressed genes are mainly located in the apical part of cell, synaptic membrane, collagen−containing extracellular matrix and postsynaptic membrane. They are mainly involved in biological processes such as signal release, response to metal ions, hormone metabolic process, and neurotransmitter transport.

KEGG pathway analysis showed that *PSMD14* may play a role in HCC by participating in neuroactive ligand−receptor interaction, cAMP signaling pathway, protein digestion and absorption and bile secretion (Fig. **6F**).

## 8 DISCUSSION

The recent 2022 cancer statistics report highlights that the high recurrence and mortality rates of HCC underscore the pressing need to discover new prognostic markers.

In this study, the oncogenes in HCC were screened by CCLE, GEO and TCGA database analysis and verified by Q-PCR in 5 cases of HCC tissues and the corresponding adjacent non-tumor tissues. Finally, *ATP6AP1*, *PSMD14* and *HSP90AB1* were chosen to validate the expressions in 63 cases of HCC tissues and the corresponding adjacent non-tumor tissues. Then, we analyzed the relapse-free survival rate (RFS) of abnormal expression of *ATP6AP1*, *PSMD14* and *HSP90AB1* in these 63 HCC tissues and paired non-tumor tissues through five years of follow-up and in the K-M plotter database. We found the high expression of the 3 oncogenes in tumor tissue might be related to the poor RFS of HCC patients. Intriguingly, while the survival analysis results for *ATP6AP1* were not statistically significant (*P* > 0.05), the trend indicated that high expression of *ATP6AP1* was positively correlated with the recurrence rate of patients, successfully distinguishing the high-expression group from the low-expression group. At the same time, in the GEPIA database, the high expression of *ATP6AP1* is inversely proportional to the ten-year Overall Survival (OS) and is statistically significant (logrank *p* = 0.027). The RFS of clinical patients in our study was not associated with the expression of *ATP6AP1*, potentially due to the limited number of cases and insufficient follow-up duration. Whether considering the K-M plotter, GEPIA database, or the clinical data we acquired, high expression of *PSMD14* is associated with a higher recurrence rate of HCC patients. Expression of *HSP90AB1* is significantly related to patient RFS in the K-M plotter database, but this correlation was not observed in our clinical data, which may also be attributed to the smaller sample size and shorter follow-up time. In addition, the mutation rate of *PSMD14* in HCC patients was 2.12%. The most common type of *PSMD14* mutation in HCC is deep deletion. Mutations in the *PSMD14* gene can also significantly affect the prognosis of patients with HCC. The KEGG pathway enrichment analysis suggested that *PSMD14* might play a role in HCC by participating in neuroactive ligand−receptor interaction, cAMP signaling pathway, protein digestion and absorption and bile secretion.

*PSMD14* (proteasome 26S subunit, non-ATPase 14) encodes a component of the 26S proteasome, which catalyzes the degradation of ubiquitinated intracellular proteins. It has been reported that this gene facilitates the progression of metastasis in esophageal cancer [56]. There were also reports that overexpression of *PSMD14* promoted the progress of ovarian cancer and gastric cancer [25, 29, 57]. *ATP6AP1* (ATPase H+ transporting accessory protein 1) encodes a component of a multi-subunit enzyme, V-ATPase, that mediates acidification of organelles in eukaryotic cells, which is necessary for intracellular processes such as protein sorting, zymogen activation, and receptor-mediated endocytosis. Pareja F. *et al.* [18] and Sekimizu M. *et al.* [58] reported that this gene is an oncogenic driver of granular cell tumors. Jansen EJ depicted that *ATP6AP1* deficiency was capable of leading to immunodeficiency with hepatopathy [16]. Both Tian Y. *et al* and Wang J. *et al.* reported that *ATP6AP1* was related to the progress and prognosis of breast cancer and might become a biomarker of breast cancer prognosis [59, 60]. *HSP90AB1*(heat shock protein 90 alpha family class B member 1) encodes a member of the heat shock protein 90 family, involved in signal transduction, protein folding and degradation and morphological evolution. In recent years, studies concerning *HSP90AB1* have identified its function in neoplasms. It was shown that the overexpression of this gene was likely to be associated with poor prognosis in lung cancer [61]. Besides, *HSP90AB1* plays an important role in promoting tumor progression in melanoma [62], breast cancer [63], gastric cancer [64], and head and neck squamous cell carcinoma [45]. Although the genes above have been reported in various tumors, the expression and the biological function of HCC are still unclear.

## CONCLUSION

In this study, we validated the high expression of *ATP6AP1, PSMD14* and *HSP90AB1* in HCC tissue and the aberrant expression of *PSMD14* in tumor tissue related to the poor prognosis. However, we didn’t reveal the biological functions or the specific mechanisms through which these genes impact the recurrence in HCC patients. Further investigations are needed in the future.

## Figures and Tables

**Fig. (5):**
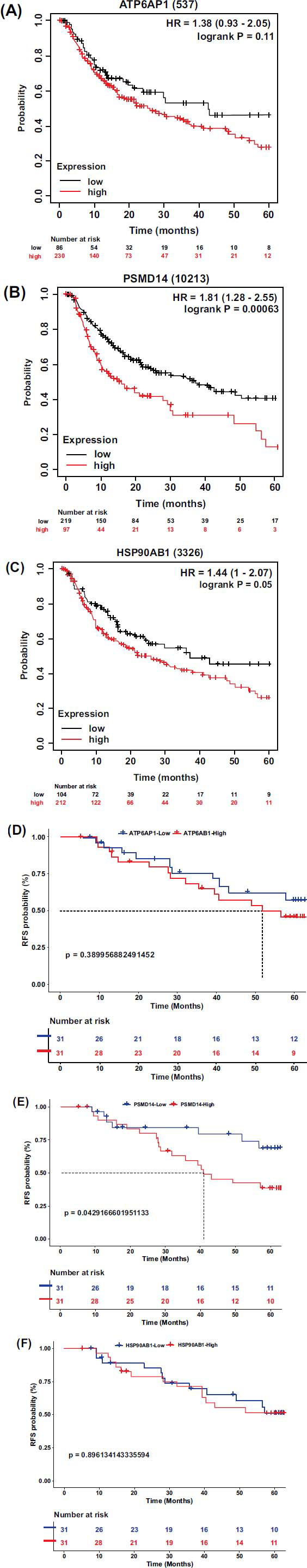
Kaplan–Meier curves of relapse free survival (RFS) comparing the high and low expression of (**A**) *ATP6AP1*, (**B**) *PSMD14* and (**C**) *HSP90AB1* in liver cancer in the Kaplan–Meier plotter databases (n=316). The Kaplan–Meier curves of RFS with high expression and low expression of (**D**) *ATP6AP1*, (**E**) *PSMD14* and (**F**) *HSP90AB1* were obtained through a follow-up of 62 cases of HCC tissue previously taken.

**Table 1:** The primers of the 8 genes and *GAPDH*

**Gene**	**Primer**	
NUP37	Forward	TGCAACCACTGGTTATCCTGGC
	Reversed	AGTTCGATGCCAGGACAGTCCA
*ATP6AP1*	Forward	CCTTCTGGAATGACTCCTTTGCC
	Reversed	GAGCCATTGCTGTGGACTTCGA
PRIM1	Forward	TATCGCTGGCTCAACTACGGTG
	Reversed	CACTCTGGTTGTTGAAGGATTGG
*HSP90AB1*	Forward	CTCTGTCAGAGTATGTTTCTCGC
	Reversed	GTTTCCGCACTCGCTCCACAAA
TUBB	Forward	CTGGACCGCATCTCTGTGTACT
	Reversed	GCCAAAAGGACCTGAGCGAACA
FANCI	Forward	GCAAGCTGATGTTCGACTCATGC
	Reversed	AGGCAGCAGATCAGGTTTTGGC
*PSMD14*	Forward	GTCAGTGTGGAGGCAGTTGATC
	Reversed	CCACACCAGAAAGCCAACAACC
EXO1	Forward	TCGGATCTCCTAGCTTTTGGCTG
	Reversed	AGCTGTCTGCACATTCCTAGCC
GAPDH	Forward	GTCTCCTCTGACTTCAACAGCG
	Reversed	ACCACCCTGTTGCTGTAGCCAA

**Table 2:** Kyoto encyclopedia of genes and genomes (KEGG) pathway analysis of common overexpressed DEGs between HCC cell lines and HCC tissues.

**Pathway**	**ID**	**Count**	***P* value**	**Genes**
Cell cycle	hsa04110	39	3.95E-19	ESPL1/DBF4/CDKN2A/CDKN2C/E2F3/E2F5/YWHAZ/YWHAB/YWHAG/TTK/RAD21/RBX1/CCNA2/PCNA/CCNE2/CDC7/CDC6/CCNB1/ CCNB2/PLK1/CDC45/CDK1/CDK4/CDC25B/PTTG1/MAD2L1/MAD2L2/ CHEK1/CHEK2/CDC23/CDC20/SFN/ORC3/MCM7/MCM2/MCM3/MCM5/MCM6/BUB1B
DNA replication	hsa03030	16	3.42E-11	FEN1/RNASEH2A/RPA1/PCNA/POLA1/POLE2/PRIM1/PRIM2/RFC3/RFC4/RFC1/MCM7/MCM2/MCM3/MCM5/MCM6
p53 signaling pathway	hsa04115	15	0.000013	GTSE1/AIFM2/CDKN2A/TP53I3/CCNE2/RRM2/CCNB1/CCNB2/CDK1/CDK4/RFWD2/ CHEK1/ CHEK2/SFN/GORAB
Oocyte meiosis	hsa04114	20	3.22E-05	ESPL1/PPP1CA/AURKA/FBXO5/YWHAZ/YWHAB/YWHAG/RBX1/CCNE2/CCNB1/CCNB2/PLK1/CDK1/ADCY6/PTTG1/MAD2L1/MAD2L2/CDC23/CDC20/PPP1CC
Cellular senescence	hsa04218	22	0.000139	HRAS/RAD1/PPP1CA/NRAS/CDKN2A/E2F3/E2F5/RAD9A/FOXM1/PIK3R3/CCNA2/CCNE2/CAPN2/CCNB1/CCNB2/RRAS/TSC1/CDK1/ CDK4/ CHEK1/CHEK2/PPP1CC
Gap junction	hsa04540	15	0.000151	HRAS/NRAS/TUBA4A/GJA1/TUBB3/PDGFA/TUBA1B/TUBA1C/TUBB2A/GNAI1/TUBB4B/TUBB/CDK1/ADCY6/PLCB1
Fanconi anemia pathway	hsa03460	11	0.00023	RMI2/RMI1/BRIP1/FANCI/FANCG/BLM/BRCA1/UBE2T/RPA1/RAD51C/USP1
Mismatch repair	hsa03430	7	0.000238	EXO1/RPA1/PCNA/RFC3/RFC4/RFC1/MSH2
Homologous recombination	hsa03440	9	0.000474	TOPBP1/BRIP1/BLM/BRCA1/RPA1/BARD1/RAD51C/RAD54L/SHFM1
Progesterone -mediated oocyte maturation	hsa04914	15	0.000566	MAPK9/AURKA/PIK3R3/CCNA2/GNAI1/CCNB1/CCNB2/PLK1/CDK1/CDC25B/ADCY6/MAD2L1/MAD2L2/CDC23/HSP90AB1
Lysosome	hsa04142	17	0.000741	LAPTM4B/SORT1/GLA/NEU1/LAMP3/AGA/SGSH/AP3S1/CLN3/NAGPA/ATP6AP1/AP1M2/GNS/CTSA/CTSV/CTSC/SLC17A5
Human T-cell leukemia virus 1 infection	hsa05166	25	0.000925	HRAS/ESPL1/MAPK9/NRAS/CDKN2A/CDKN2C/E2F3/MAP3K1/PIK3R3/CCNA2/MSX1/CCNE2/CCNB2/XPO1/FDPS/CDK4/ADCY6/PTTG1/MAD2L1/CHEK1/CHEK2/CDC23/CDC20/TRRAP/BUB1B
Proteasome	hsa03050	9	0.000975	SHFM1/PSMD14/PSMA4/PSMB4/PSMC4/PSMD1/PSMD2/PSMD4/PSME3
Pathogenic *Escherichia coli* infection	hsa05130	10	0.001128	ARPC2/TUBA4A/TUBB3/YWHAZ/TUBA1B/TUBA1C/TUBB2A/TUBB4B/ARPC1B/TUBB
RNA transport	hsa03013	20	0.001423	POP5/THOC2/THOC1/UPF2/TACC3/RPP40/EIF3D/EIF3H/NUP155/NUP205/XPO1/XPO5/DDX20/XPOT/NUP37/NUP62/NXT2/EIF2B1/NUPL2/KPNB1

**Table 3:** The enriched KEGG pathways for eight candidate genes.

**Gene**	**KEGG Pathway**
ATP6AP1	Lysosome
EXO1	Mismatch repair
FANCI	Fanconi anemia pathway
HSP90AB1	Progesterone-mediated oocyte maturation
NUP37	RNA transport
PRIM1	DNA replication
PSMD14	Proteasome
TUBB	Gap junction

## Data Availability

The datasets generated during and/or analyzed during the current study are available from the corresponding author upon reasonable request.

## LIST OF ABBREVIATIONS

CCLE: Cancer Cell Line Encyclopedia
DEGs: Differentiated Expressed Genes
DFS: Disease-free Survival
DUB: Deubiquitinase
EGA: European Genome-phenoome Archive
GEO: Gene Expression Omnibus
GEPIA: Gene Expression Profiling Interactive Analysis
GO: Gene Ontology
GSEA: Gene Set Enrichment Analysis
GTEx: Genotype-Tissue Expression
HCC: Hepatocellular Carcinoma
HPA: Human Protein Atlas
KEGG: Kyoto Encyclopedia of Genes and Genomes
OS: Overall Survival
PEI: Percutaneous Ethanol Injection
Q-PCR: Quantitative Polymerase Chain Reaction
RFA: Radiofrequency Ablation
RFS: Relapse-free Survival
RMA: Robust Multi-array Average
TACE: Transarterial Chemoembolization
TCGA: The Cancer Genome Atlas
